# Evaluation of the CareStart™ glucose-6-phosphate dehydrogenase (G6PD) rapid diagnostic test in the field settings and assessment of perceived risk from primaquine at the community level in Cambodia

**DOI:** 10.1371/journal.pone.0228207

**Published:** 2020-01-31

**Authors:** Bertha Wojnarski, Chanthap Lon, Darapiseth Sea, Somethy Sok, Sabaithip Sriwichai, Soklyda Chann, Sohei Hom, Threechada Boonchan, Sokna Ly, Chandara Sok, Samon Nou, Pheaktra Oung, Nareth Kong, Vannak Pheap, Khengheang Thay, Vy Dao, Worachet Kuntawunginn, Mitra Feldman, Panita Gosi, Nillawan Buathong, Mali Ittiverakul, Nichapat Uthaimongkol, Rekol Huy, Michele Spring, Dysoley Lek, Philip Smith, Mark M. Fukuda, Mariusz Wojnarski

**Affiliations:** 1 Armed Forces Research Institute of Medical Sciences, Bangkok, Thailand; 2 The George Washington University, School of Nursing, Washington, DC, United States of America; 3 Ministry of National Defense, Department of Health, Phnom Penh, Cambodia; 4 Chenda Polyclinic (CPC), Phnom Penh, Cambodia; 5 National Center for Parasitology, Entomology and Malaria Control, Phnom Penh, Cambodia; 6 School of Public Health, National Institute of Public Health, Phnom Penh, Cambodia; Academic Medical Centre, NETHERLANDS

## Abstract

**Background:**

Primaquine is an approved radical cure treatment for *Plasmodium vivax* malaria but treatment can result in life-threatening hemolysis if given to a glucose-6-phosphate dehydrogenase deficient (G6PD*d*) patient. There is a need for reliable point-of-care G6PD diagnostic tests.

**Objectives:**

To evaluate the performance of the CareStart™ rapid diagnostic test (RDT) in the hands of healthcare workers (HCWs) and village malaria workers (VMWs) in field settings, and to better understand user perceptions about the risks and benefits of PQ treatment guided by RDT results.

**Methods:**

This study enrolled 105 HCWs and VMWs, herein referred to as trainees, who tested 1,543 healthy adult male volunteers from 84 villages in Cambodia. The trainees were instructed on G6PD screening, primaquine case management, and completed pre and post-training questionnaires. Each trainee tested up to 16 volunteers in the field under observation by the study staff.

**Results:**

Out of 1,542 evaluable G6PD volunteers, 251 (16.28%) had quantitative enzymatic activity less than 30% of an adjusted male median (8.30 U/g Hb). There was no significant difference in test sensitivity in detecting G6PD*d* between trainees (97.21%), expert study staff in the field (98.01%), and in a laboratory setting (95.62%) (p = 0.229); however, test specificity was different for trainees (96.62%), expert study staff in the field (98.14%), and experts in the laboratory (98.99%) (p < 0.001). Negative predictive values were not statistically different for trainees, expert staff, and laboratory testing: 99.44%, 99.61%, and 99.15%, respectively. Knowledge scores increased significantly post-training, with 98.7% willing to prescribe primaquine for *P*.*vivax* malaria, an improvement from 40.6% pre-training (p < 0.001).

**Conclusion:**

This study demonstrated ability of medical staff with different background to accurately use CareStart™ RDT to identify G6PD*d* in male patients, which may enable safer prescribing of primaquine; however, pharmacovigilance is required to address possible G6PD*d* misclassifications.

## Introduction

Malaria elimination remains a challenge in resource-limited settings such as Cambodia, despite 10 years of progressive gains towards this goal. Among the different malaria species in Southeast Asia, *Plasmodium vivax* (*Pv*) is most prevalent [1]. *Pv* treatment poses unique challenges for malaria elimination because it remains dormant in the liver as a hidden reservoir and is responsible for multiple relapses. Primaquine (PQ) is currently the only US Food and Drug Administration (FDA) approved drug available in Southeast Asia for radical cure that eliminates *Pv* from the liver [2]. However, a radical cure dose, 30 mg of PQ for 14 days, can result in significant hemolytic toxicity if incorrectly prescribed to a glucose-6-phosphate dehydrogenase deficient (G6PD*d*) patient [2,3]. Similar concerns of hemolytic toxicity exist for a 15 mg daily dose for 14 days, which is currently recommended per the national malaria treatment guidelines in Cambodia. A single dose of tafenoquine (TQ), which was approved by the FDA in 2018 for treatment of latent *Pv* infections, is also associated with the same hemolytic toxicity in G6PD*d* individuals [4]. Therefore, G6PD screening and effective risk communication by providers will be required prior to PQ and TQ administration [5].

PQ is not widely used in Cambodia due to unavailability of suitable G6PD screening tests, which negatively impacts the progress of malaria elimination in the region. The reduction in malaria cases observed in Cambodia over the last decade is primarily due to the lower burden of *P*. *falciparum* (*Pf*) malaria [2,6]. Recently, G6PD point-of-care tests have become commercially available but provider training on G6PD*d* diagnosis and the use of G6PD rapid diagnostic tests (RDTs) at the community level is poorly defined [7]. In order to eliminate *Pv* by 2025, the goal set by the Cambodian government, the implementation of screening for G6PD*d* at the community level will be required [8].

G6PD*d* affects close to 400 million individuals worldwide [9]. In Cambodia, the prevalence of G6PD*d* was estimated at 13–26% in males and 3–4% in females [6,10,11]. Therefore, inexpensive, reliable, and easily accessible G6PD*d* screening tests are needed for a safe administration of 8-aminoquinoline drugs, such as PQ or TQ [1,12,13]. However, few studies examined and validated G6PD screening tests at the point-of-care in the village where patients usually seek care for their malaria symptoms. A 2010 study using the 2^nd^ generation CareStart^TM^ G6PD RDT kit demonstrated a sensitivity of 68% (95% CI: 58–77) and specificity of 100% (95% CI: 95–100%) when the test was performed by lab technicians [14]. This study evaluated the 3^rd^ generation CareStart^TM^ G6PD RDT and the impact of two-day training on test performance and knowledge gain around primaquine induced hemolysis. There are no prior studies that evaluated readiness to incorporate G6PD testing at the community level in Cambodia, or assessed the perceptions of healthcare providers on the risks of primaquine treatment in the context of commercially available G6PD screening tests.

According to the WHO, G6PD point-of-care tests must be able to detect G6PD*d* in males with less than 30% of enzymatic activity, and have test sensitivity of at least 95% to guide primaquine treatment [4,13]. Despite positive results from laboratory settings, there is still insufficient data to determine if the CareStart™ RDT could achieve the WHO required thresholds for test sensitivity and negative predictive values, when the test is performed in field settings by the health care workers (HCWs) and/or village malaria workers (VMWs) [15]. Given the potential risks associated with misdiagnosed G6PD status, the use of G6PD RDT in a community setting will require community engagement and a careful assessment of risks [13]. The WHO and National Center for Parasitology, Entomology and Malaria Control (CNM) cautioned that the deployment of RDTs for G6PD screening should be piloted in real-life field settings, before recommending larger scale-up of the program in Cambodia as a national policy [16]. Therefore, the purpose of this study was to assess the performance of the CareStart™ G6PD RDT in field settings in the hands of trainees with different background, and compare these with the results obtained by trained study staff in both field and laboratory settings. User perceptions about the risks and benefits of PQ treatment guided by RDT results were also assessed.

## Methods

Study specific aims were to: 1) develop training materials to improve knowledge and acceptability of PQ use by trainees; 2) assess the performance of the CareStart™ for G6PD*d* screening in the field settings; 3) compare HCWs/VMWs results to those obtained from trained study staff (expert observers) in both field and laboratory settings; 4) evaluate trainee’s ability to correctly perform and interpret G6PD RDT results; 5) and assess user perceptions about the risks and benefits of PQ treatment and their willingness to prescribe PQ based on the results obtained by RDT.

### Ethics statement

The funding support was provided by Defense Malaria Assistance Program (DMAP). This study was approved by the National Ethics Committee for Health Research (NECHR) of the Ministry of Health of Cambodia and the WRAIR IRB under protocol number WR2478. In addition, the protocol was submitted for review by the George Washington University IRB to assure compliance with their institutional policies. Finally, the protocol was reviewed by the Human Subjects Division of the Centers for Disease Control and Prevention, for a non-engaged determination. There were no unanticipated problems involving risks to volunteers or others, and no significant deviations were noted. Informed consent was obtained from all volunteers (G6PD test volunteers and trainees who participated in G6PD test evaluations).

### Study design

**Fig 1:**
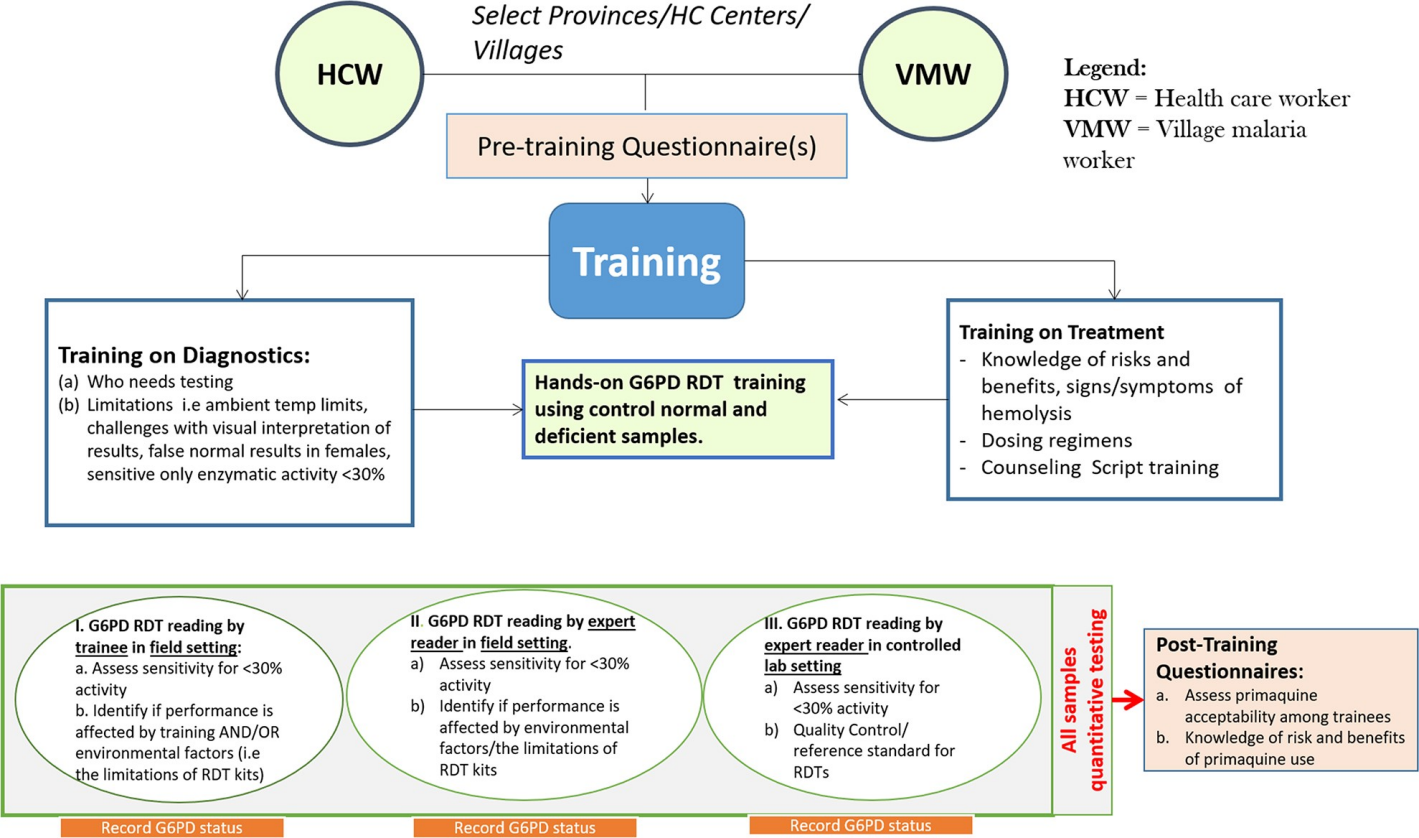
Project overview.

This study was conducted in 84 Cambodian villages of the Oddar Meanchey province where the CNM initiated deployment of single dose PQ in 2019 and also has plans to deploy radical cure in the near future. The study was conducted in 2 Phases. Phase 1 focused on training of HCWs/VMWs utilizing didactic PowerPoint presentations and a counseling script, question and answer sessions, and hands-on session on the use of G6PD RDTs. During this two-day workshop, trainees were evaluated on the correct use of four to six RDTs of the same brand (CareStart^TM^) with commercially available G6PD standards (deficient, normal, and intermediate control samples) obtained from WellsBio (CareUS^TM^ G6PD RDT controls, catalogue number RGC-N10082). All trainees were required to pass a written knowledge test at the end of 2-day training before they qualified to test volunteers in the field. Of 105 trainees, 101 completed Phase 1. In Phase 2, each trainee conducted G6PD screening of up to 16 volunteers in the field settings, and completed field surveys based on their field experience with the test (Fig 1).

### Study sites

Trainees were enrolled from the district referral hospital, 11 health centers, and local health posts located at or near the Anlong Veng and Trapang Prasath districts, in Oddar Meanchay Province. The healthcare facilities where this study took place have 33 HCWs and 174 VMWs who are responsible for more than 50 villages. The regional ethnic composition is primarily Khmer, agrarian community where 75% are farmers and loggers [17]. The majority of persons residing in the community have a primary (Grade 1–6) or secondary (Grade 7–9) education and 81% of those 15 years or older are literate [18].

### Study population

#### Experts/Observers

*Definition*: Research study staff with prior experience in using G6PD tests

Trainee volunteers (HCWs and VMWs)

*Definition*: Nurses, lab technicians, health center staff and village malaria workers who signed an informed consent form to be evaluated on their ability to interpret G6PD RDT tests results

#### G6PD test volunteers

*Definition*: Khmer speakers who live in the community where the study was conducted, who signed an informed consent form to have their G6PD status assessed by available diagnostic tests

*Inclusion criteria*: Healthy male volunteers at least 18 years of age, without acute symptoms, afebrile and self-reported wellbeing

*Exclusion criteria*: Patients with acute illness requiring hospitalization and/or streatment

Females were excluded as their G6PD status cannot be reliably identified with CareStart RDTs [15]. Only quantitative tests can identify G6PD deficient females with activity levels between 30–70% [13,19].

### Sample size and power calculation for G6PD test volunteers

Previous studies conducted in Cambodia reported average G6PD prevalence in males around 15% [5]. The sensitivity of G6PD RDT tests to detect G6PD*d* (<30% enzyme activity) in ideal lab conditions was estimated to be 99 to 100% [3]. Therefore, to detect a difference in the test sensitivity from 99% (ideal) to 95% (trainees), a sample size of 1,560 and 15% prevalence of G6PD*d* was required to achieve 80% power using 5% two-sided test.

### Statistical methods

The data were retrieved from case report forms (CRF) and analyzed using Stata 15.0 (Statacorp, College Station, TX), SPSS and Graphpad Prism 7. A p-value < 0.05 indicated statistically significant differences. Sensitivity, specificity, positive predictive value (PPV), and negative predictive value (NPV) for detecting G6PD deficiency were calculated against the population-adjusted male median of 30% enzymatic activity on a reference quantitative test. Descriptive, inferential statistics, and Cochran’s Q test were used to analyze the data. Paired t-test was used to compare the mean values on samples from the same volunteer. The percentage of correctly performed and/or interpreted tests was recorded. G6PD status that could not be classified (volunteer could not make a call about the color change) did not count as failure on test performance but the percentage of tests without status classification was reported within each group. Missing data was recorded as a failed result.

### Recruitment and informed consent

The Armed Forces Research Institute of Medical Sciences (AFRIMS) team in Cambodia advertised the screening through VMWs, with the authorization of the village chiefs and malaria supervisors. Meetings were held with the Ministry of Public Health and CNM to coordinate the participation of the VMWs. Volunteers for G6PD testing were recruited from villages and the health centers. Trainees were recruited from their work stations based on proximity to the training sites in Oddar Meanchey. All trainees were required to obtain a signed supervisor’s note before participation in the study. Written Informed Consent Form (ICF) was obtained from all volunteers prior to study participation. The ICFs were translated into the local language (Khmer).

### Training of HCWs and VMWs

Standardized and IRB-approved training materials were developed for a 2-day workshop, focusing on G6PD screening and PQ treatment regimens to include standardized counseling script (S1 File). Other topics covered in the training included G6PD RDT test limitations, dose of PQ for *Pf* vs. *Pv*, as well as risks and benefits of PQ treatment. All study material translations were authenticated and certified. The training was conducted in Khmer language.

The first day of training consisted of theoretical training covering topics on G6PD deficiency, screening and limitations of test results, PQ associated risks and benefits, and how to recognize PQ induced hemolysis. The current treatment guidelines for malaria in Cambodia were also reviewed. A practical hands-on training was conducted on the second day of the workshop. Control samples of known G6PD activity (normal, deficient, and intermediate) were purchased from WellsBio for the hands-on training sessions. Each trainee who passed the knowledge test was assigned to screen up to 16 volunteers in the field. An expert observer (trained study staff) was assigned to each testing site on the days of screening to evaluate the procedures in the field settings. A checklist was used to document testing procedures and recording of results (S1 Table).

### G6PD testing

#### Sample collection

All blood samples were obtained from December 2017 to March 2018. Two milliliters of venous blood was used to measure a complete blood count (CBC), to process G6PD RDT (Access Bio. Inc., New Jersey, USA) testing in the lab, and to complete confirmatory quantitative G6PD testing (Pointe Scientific, Inc. MI, USA). The blood samples for CareStart™ RDT testing in the field were collected under sterile techniques following finger pricks, 2 μl of blood was added into a sample well, followed by two drops of assay buffer, and results were read at 10 minutes, following the manufacturer’s instructions. Blood samples for reference G6PD quantitative testing were collected in ethylenediaminetetraacetic acid (EDTA) treated tubes (BD Vacutainer, USA) and stored at 4°C for a maximum of 4 h between collection time and laboratory analysis of G6PD activity.

#### Blinding

Trainees and expert study staff were blinded to the quantitative results until G6PD RDT results were recorded in the Data Collection Form (DCF) and could no longer be changed. Trainees were also blinded to interpretation of G6PD test results by trained study staff and experts in the lab, until results from both were recorded and locked.

#### G6PD test interpretation

A spectrophotometer (Shimadzu UV 1800 series, Shimadzu, Kyoto, Japan) was used to measure the change in absorbance at 340 nm over 5 min, using Temperature-Controlled Cell Positioner, CPS-100, Shimadzu, Japan. Normal and deficient G6PD activity controls from Trinity Biotech (catalogue numbers G6888 and G5888) were run in duplicate every morning that volunteers were being enrolled. Sample testing was done if all controls had values within a predefined activity range provided by the manufacturer. Duplicates for which the measurement values differed by ≥ 0.5 U/g Hb were retested.

Results from CareStart^TM^ G6PD RDT obtained by trainees were compared against those obtained by trained study staff in the field and experts in the lab. G6PD RDT status determination was based on color change in the reading window, following the manufacturer recommendations, with no color change classified as deficient and change to purple classified as normal [13,14]. The CareStart™ G6PD RDT color chart, developed by AccessBio, was utilized to aid in color change interpretation. For samples with very subtle purple color, or when change in color was not clear, volunteers were asked to select “unclassified’ for the status interpretation.

All blood samples were analyzed on the same day of collection. All results from RDTs were compared against the G6PD spectrophotometric quantitative analysis (Pointe Scientific, Inc., MI, USA), which was performed in AFRIMS research laboratory, in Anlong Veng Referral Hospital, Cambodia. G6PD enzymatic activity of <30% of the adjusted male median (AMM) was the threshold for defining G6PD***d*** in male volunteers, consistent with the WHO guidelines [19]. AMM for the study population was calculated by excluding males with severe G6PD deficiency, defined as having enzymatic activity ≤ 10% of the median for all males in the study population.

#### Result notification and recording of G6PD status

Volunteers were not informed of their G6PD test results until their G6PD status was confirmed by quantitative test after all samples had been tested. The G6PD test volunteers were also provided with a G6PD Alert Card. The card included study staff phone number in case of questions about test results. Volunteers were asked to present the card during their clinic visits and hospital admissions. The card indicated volunteer’s G6PD test results as either normal or deficient. Volunteers with borderline test results (30–40% G6PD activity) were informed that their G6PD status could not be classified, received additional counseling, and were advised to have the test repeated.

### Assessment of knowledge and perceptions about primaquine risks and benefits

A standardized questionnaire was developed to assess knowledge, acceptability, and risk perceptions of the trainees in regards to PQ use and CareStart RDT testing. Questionnaires were completed both pre- and post-training. Structured and semi-structured questions were utilized, with varying responses, such as *not willing* to *willing*, *not comfortable* to *comfortable*, or *not reliable* to *reliable*. The percentage of trainees willing to use G6PD RDT as a routine component of malaria case management was evaluated. Additional questions on demographics included participant’s age, occupation, gender, years of experience, and level of education.

The pre-training questionnaires consisted of 44 questions, 29 of the questions focused on knowledge assessment. Each correct answer received a score of 1. Additional questions assessed a trainee’s willingness to prescribe PQ, and their understanding of treatment risks and benefits, and test limitations. Responses by trainees were rated on a 7-point Likert scale which was reduced to a 5-point scale for analysis. For interpretation of results, a score of 1 and 2, and 6 and 7 were grouped together, unless specified differently in the results section. Perceptions on the risks and benefits were measured by assessing trainee responses such as “*not comfortable = 1*” to “*comfortable = 7*.” Similarly, the post-training questionnaire consisted of 43 questions, including the same 29 knowledge questions from pre-training questionnaire and additional acceptability and risk perception questions. Post-training questionnaires were completed immediately post training on Day 2, and Week 2, Week 4, Week 6, and Week 8. Trainees with knowledge scores below 100% received additional instruction. The assessment of feasibility of deploying G6PD RDTs was quantified by the percentage of HCWs and VMWs willing to prescribe PQ based on the RDT results alone, after explanation of test limitations.

## Results

A total of 105 trainees and 1,543 G6PD test volunteers enrolled in the study from 18 December 2017 to 5 March 2018; however, 4 trainees were withdrawn as they did not complete the training, and 1 G6PD test volunteer was withdrawn due to unsuccessful blood collection. The majority (53.4%) of trainees reported minimal exposure and knowledge about primaquine, with 95% of volunteers not having dispensed or prescribed PQ prior to the workshop (Table 1).

**Table 1:** Baseline demographics.

**Age in years, mean (SD)**	**Trainees**	38.14 (12.47)
**Sex, n (%)**	Male	44 (43.56)
	Female	57 (56.44)
**Occupation/Profession, n (%)**	Village malaria worker	74 (73.3)
	Nurse	21 (20.8)
	Other	6 (5.9)
**Education, n (%)**	Primary school	45 (44.55)
	Secondary school	34 (33.66)
	High school	12 (11.88)
	College	6 (5.94)
	Post-graduate	4 (3.96)
**Experience working at the current job, n (%)**	< 1 year	18 (17.82)
	1 to < 3 yrs.	20 (19.8)
	3 to < 5 yrs.	18 (17.82)
	5 yrs. or more	45 (44.55)
**Work setting, n (%)**	Hospital	1 (0.99)
	Health center/post	25 (24.75)
	Community/VMW	75 (74.26)
**Heard about drug primaquine (PQ)?**	No	47 (46.53)
	Yes	54 (53.47)
**Dispensed or prescribed primaquine?**	No	95 (94.06)
	Yes	6 (5.94)
**Treated a patient with acute hemolysis or severe anemia?**	No	94 (93.07)
	Yes	7 (6.93)
**Heard of glucose-6-phosphate dehydrogenase (G6PD) deficiency?**	No	88 (87.13)
	Yes	13 (12.87)

### G6PD test results

**Fig 2:**
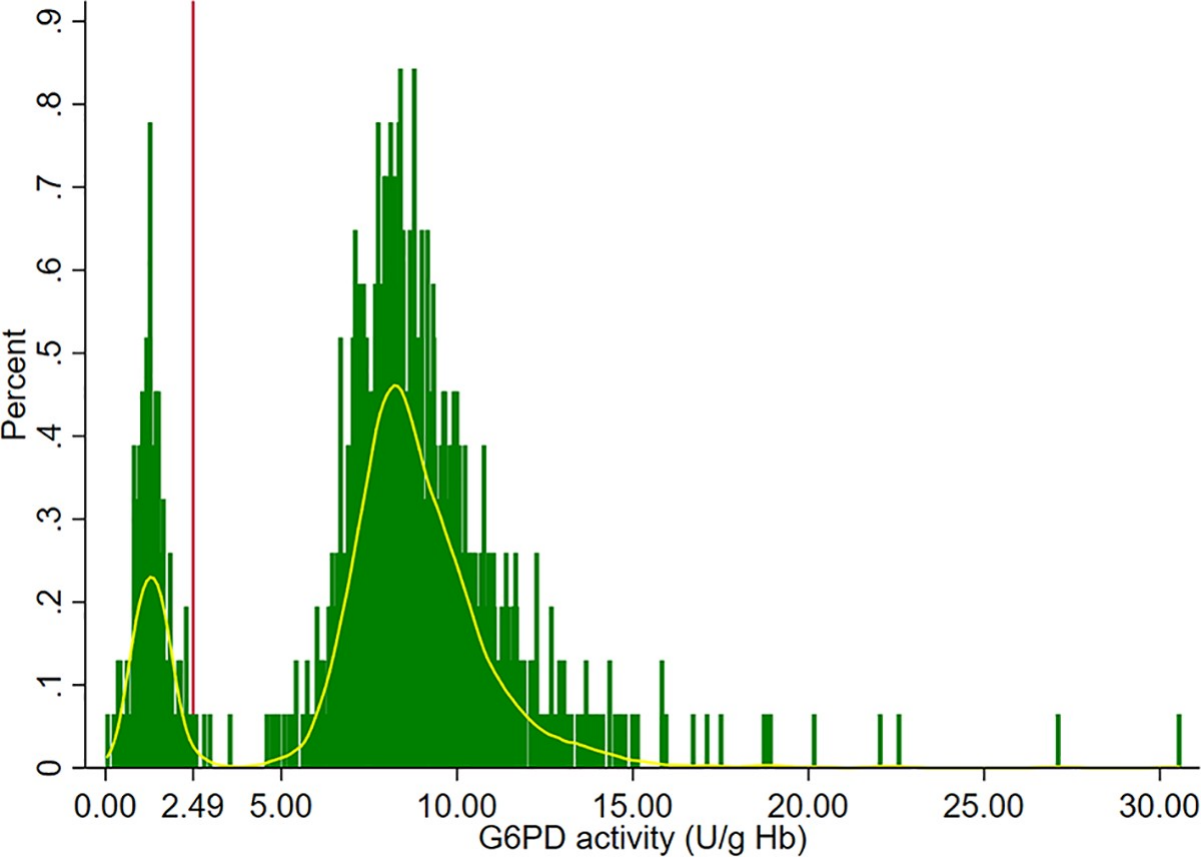
Distribution of G6PD enzymatic activity levels among male study population in Cambodia. The bimodal distribution of G6PD activity levels was observed as was expected for male volunteers enrolled in the study. There were nine volunteers whose G6PD quantitative test results ranged between 30% and 60%. Among the nine, three had borderline low G6PD activity levels (30.1%, 31.0% and 33.9%) with corresponding G6PD deficient status based on CareStart^TM^ RDT. Six volunteers whose G6PD enzymatic activity ranged between 35.7% to 59.3% had a normal RDT result.

1,542 G6PD test volunteers were included in the analysis of the G6PD population median with results showing typical bimodal distribution among the male study population (Fig 2). Adjusted male median G6PD activity was 8.30 U/g Hb (100% activity for the study population) with IQR of 7.11 to 9.58 U/g Hb. Twenty-three males (1.49%) had G6PD activities less than 10% of the population median. Volunteers who had G6PD activities <30% of the adjusted male median (2.49 u/g Hb) were classified as G6PD*d* (n = 251, 16.28%). Based on the cohort of testers and the setting at which G6PD testing was done (trainees, experts in the field, experts in the lab), the number of volunteers diagnosed as G6PD*d* ranged from 253 to 289 (Table 2). Trainees were more likely to over diagnose G6PD*d* compared to any other group, but also had the smallest number of missed G6PD deficient results, based on the 30% threshold on a reference test. From 1,542 tests, only 6 (0.38%) RDTs could not be interpreted by trainees due to an ambiguous color change. Unlike trainees, experts in the lab were able to provide RDT interpretation for all samples and there were no inconspicuous color changes reported within their group (Table 2).

**Table 2:** G6PD status classification and test performance (n = 1542).

	Trainee	Expert in Field	Expert in Lab	Quantitative test	p-value
**Status Classification**					
G6PD deficient, n (%)	289 (18.74)	270 (17.51)	253 (16.41)	251 (16.28)	<0.001^a^
G6PD normal, n (%)	1,247 (80.87)	1,271 (82.43)	1,289 (83.59)	1,291(83.72)	<0.001^a^
Incorrect (95% CI)	3.37 (2.53, 4.40) [52/1,542]	1.88 (1.26, 2.69) [29/1,542]	1.56 (1.00, 2.31) [24/1,542]	Not applicable	<0.001
Unclassified, n (%)	6 (0.39)	1 (0.06)	0 (0)	0 (0)	-
**Test Performance**					
Sensitivity (95% CI)	97.21 (94.33, 98.87) [244/251]	98.01(95.41, 99.35) [246/251]	95.62(92.29, 97.79) [240/251]	Not applicable	0.229
Specificity (95%CI)	96.51 (95.36, 97.45) [1,246/1291]	98.14 (97.25, 98.81) [1,267/1,291]	98.99(98.28, 99.46) [1,278/1,291]	Not applicable	<0.001^a^
NPV (95% CI)	99.44 (98.85, 99.77) [1,246/1253]	99.61 (99.09, 99.87) [1,267/1,272]	99.15 (98.47, 99.57) [1,278/1,289]	Not applicable	-
PPV (95% CI)	84.43 (79.73, 88.41) [244/289]	91.11 (87.06, 94.22) [246/270]	94.86 (91.37, 97.24) [240/253]	Not applicable	-

^a^ Cochran's Q test (matched sample). Post-hoc analysis was conducted with pairwise McNemar test.

Trainees and experts were provided with a reference color card to aid in color interpretation. The setting at which the testing was done had an effect on test performance as shown. Trainees had a tendency to over diagnose G6PD*d* in the field settings, reflected in the difference in test specificity between the testing cohorts and a higher percentage of test misclassifications (3.37%). A small number of RDTs (n = 7) could not be interpreted due to inconspicuous color change and these results are reported as ‘unclassified’ for G6PD status. Abbreviation; NPV: Negative predictive value; PPV; Positive Predictive value.

Test for equality of proportions of G6PD outcomes in matched samples showed significant RDT result differences between trainees, experts in field, and laboratory data when compared to the quantitative test (Cochran's Q test, p < 0.001) (Table 2). There were significant differences between RDT results by the trainees when compared to experts in the field (McNemar chi-square test, p = 0.011), with an inconsistency level greatest for trainees at 3.37% (95% CI: 2.53, 4.40) and lowest for experts in the lab at 1.56% (95% CI: 1.00, 2.31) (Table 2). There was no significant difference in the RDT sensitivity to detect G6PD deficiency against the reference quantitative test by trainees (97.21%), experts in field (98.01%), and experts in the laboratory (95.62%; Cochran's Q test, p = 0.229) (Table 2). However, among G6PD normal volunteers, there was significant difference in the RDT specificity between trainees (96.51%), experts in field (98.14%), and experts in laboratory (98.99%; Cochran's Q test, p < 0.001). Further analysis showed no difference in the specificity of RDTs by experts in the lab vs. the experts performing the test in the field (McNemar Chi-square test, p = 0.052).

### Knowledge and acceptability assessment

**Fig 3:**
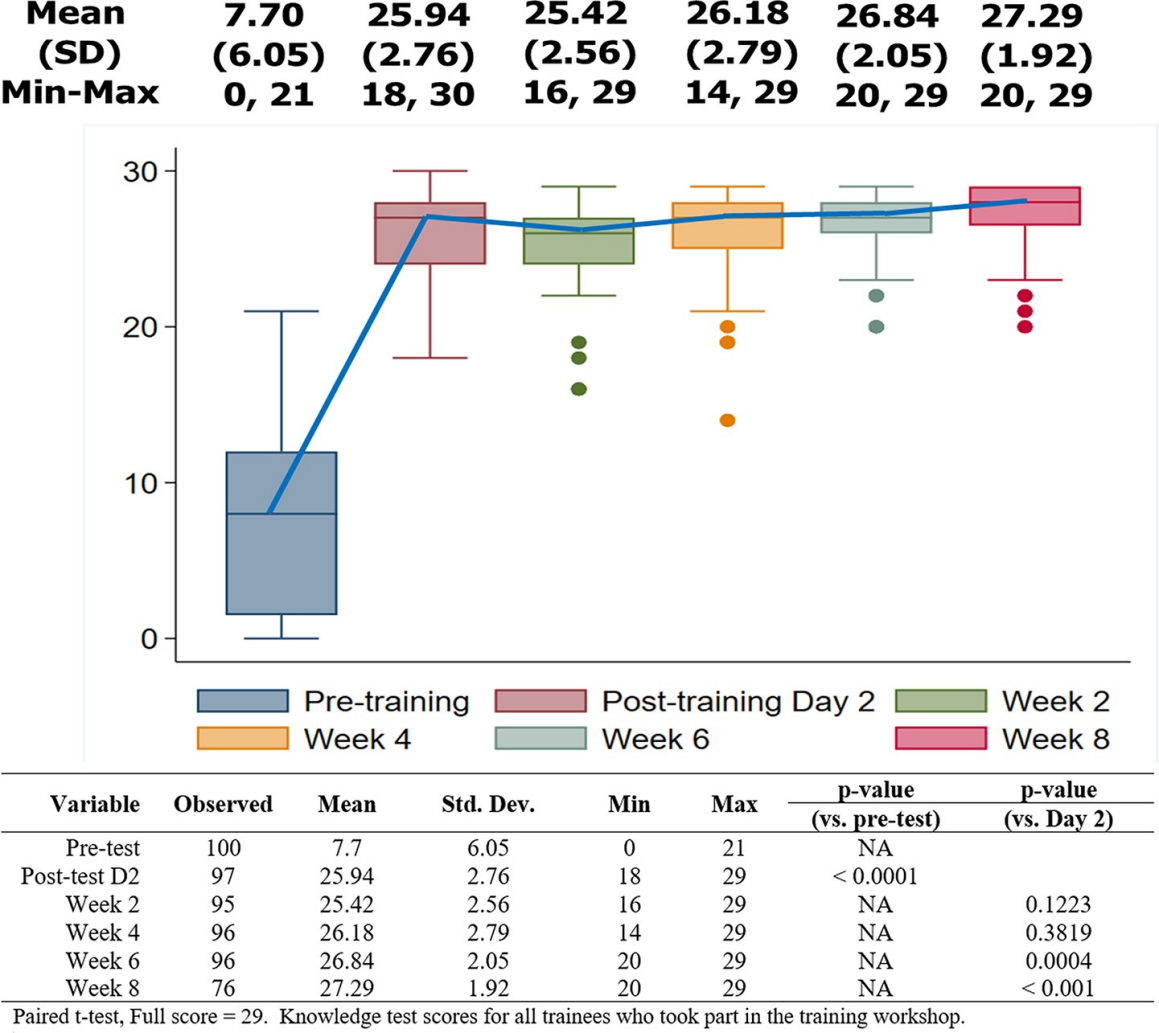
Knowledge scores for trainees who took part in the training workshop. Significant improvement in the knowledge scores was observed following training, and maintained through week 8 follow up. There was additional improvement in the knowledge scores on week 6 and week 8 follow up. The list of questions utilized to assess knowledge are included in **S2 Table**.

**Fig 4:**
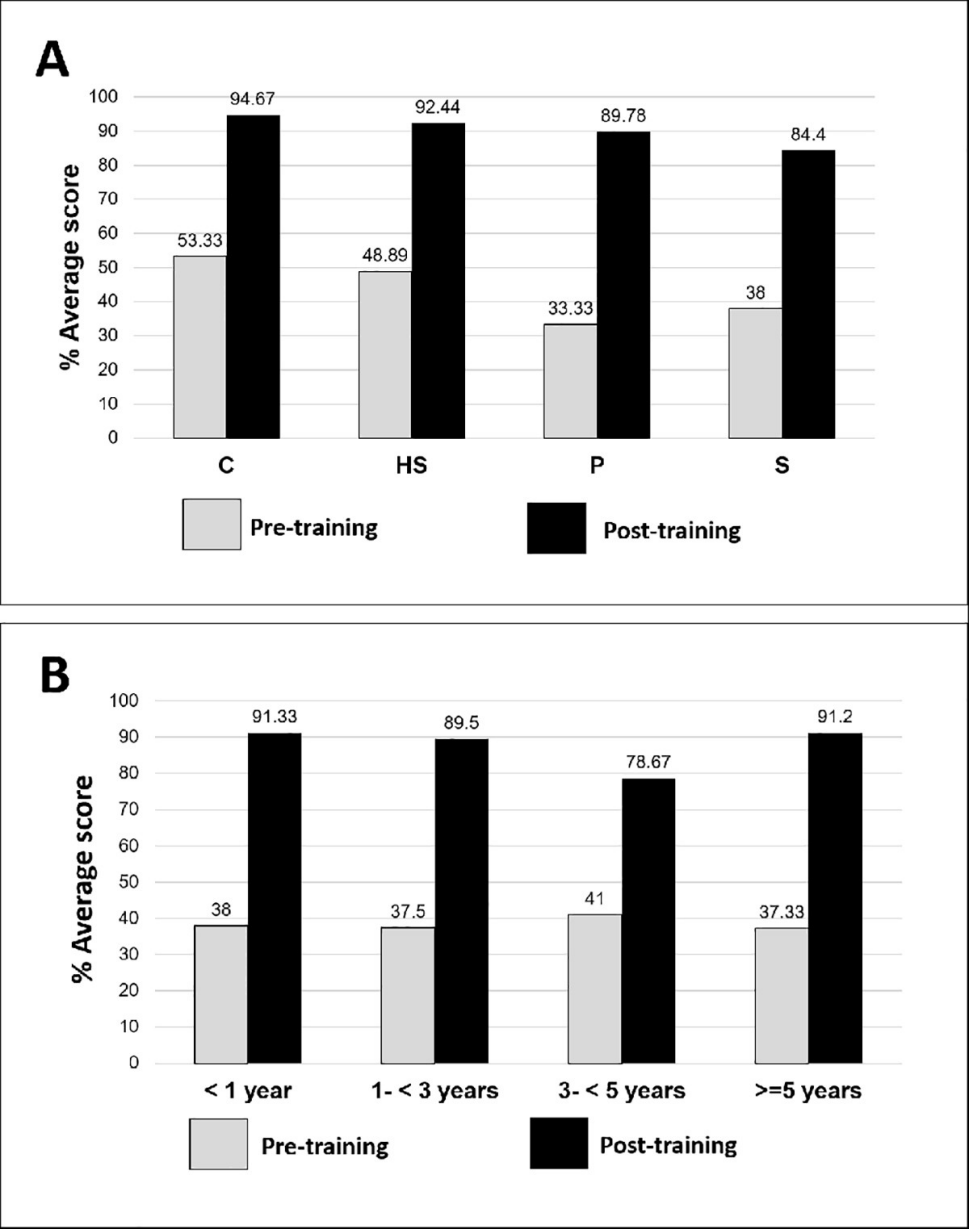
Knowledge assessment score based on highest level of education achieved by the trainees and their level of experience. Scores improved post training for all education levels: college (C), high-school (HS), primary-level education (P), and secondary-school education (S) (p<0.001) (Panel A). Scores improved post training for all levels of experience (p<0.001) (Panel B).

Trainee knowledge about G6PD testing and PQ was assessed with evaluation of covariates for their professional roles, educational levels, and years of experience at current position. At baseline, among the trainees, nurses had the highest knowledge score of 56%. However, all trainees showed improvement in their knowledge scores from pre- to post- training (p < 0.001; Fig 3), with no statistical significance in results between the groups post-training (Fig 4A and 4B). Pre-training, trainees with a college level education answered 53% of the questions correctly, as compared to 33% for those with primary level education. Post-training, VMWs with primary school level education, and 1 to 3 years of experience, had higher scores, but not significantly different from scores of VMWs with equal education, but less experience (p = 0.380). In relation to experience, there were no statistical differences in the mean knowledge scores based on the profession (p = 0.570), or education level (p = 0.380) post-training. Knowledge scores improved post-training for all education levels (p < 0.001) and for all levels of experience (p < 0.001; Fig 4A & 4B), and were maintained through follow up, with additional details provided for each question in S2 Table.

### PQ acceptability and risk perception

**Fig 5:**
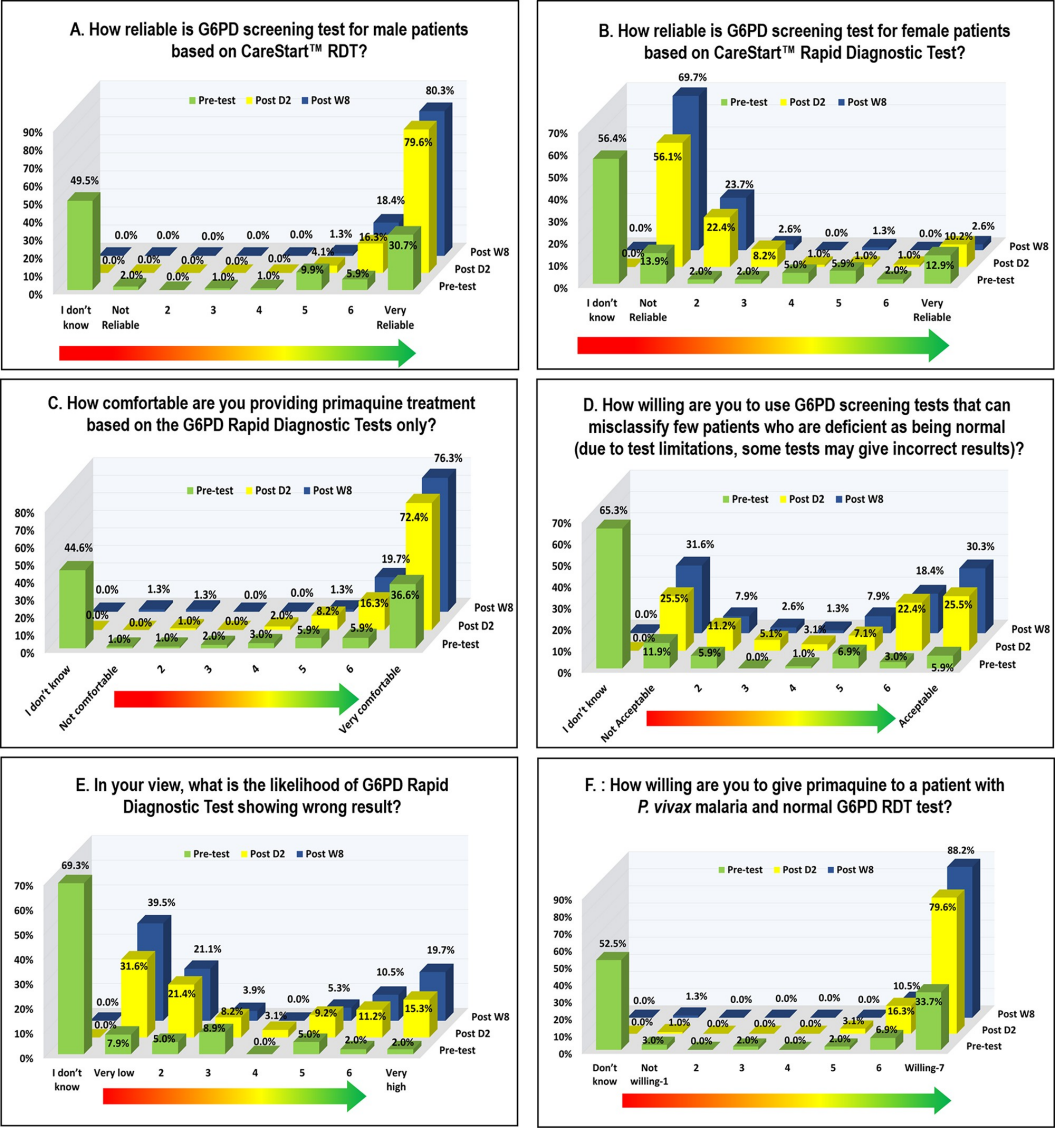
Acceptability assessment for using G6PD RDTs to guide treatment decisions with primaquine. 7-point Likert scale was used and scores of 1–2 and 6–7 were combined for classifying reliable vs. not reliable (Panel A, B), not comfortable vs. comfortable (Panel C), not acceptable vs. acceptable (Panel D), low likelihood of misclassification vs. high likelihood of misclassification (Panel E), and not willing vs. willing to give primaquine (Panel F). Stuart-Maxwell test was used to assess the difference in results between pre-training vs. Day 2, pre-training vs. Week 8, and Day 2 vs. Week 8 assessments. There was statistical difference in the responses between pre- and Day 2 follow up for all questions (p < 0.001) and pre- vs. week 8 assessments (p < 0.001), with no significant change observed between Day 2 and week 8 follow up. This highlights the positive impact of training on risk perception and acceptability of PQ, with no negative changes in perceived risk or acceptability observed with continued field experience.

This study assessed trainee acceptability to screen for G6PD*d* using the RDTs, the perception of risk for a treatment decision that was based on RDT result, and level of comfort with prescribing PQ. Overall, scores for willingness to test for G6PD*d*, and for trainees’ level of comfort in prescribing PQ, significantly improved with training (p < 0.001). At Day 2 post-training, 95.9% of the trainees stated that the kit was reliable, which was a statistically significant improvement from baseline reporting (p < 0.001; Fig 5A). There was statistically significant improvement in scores reported for the test reliability pre-training vs. Day 2 and Week 8 (p < 0.001; Fig 5A). There was also no decrease in test reliability scores between Day 2 and Week 8 follow up (p = 0.513; Fig 5A), which highlighted that with continued use of the RDTs in the field, trainees continued to report high reliability of the test in male volunteers.

Trainees’ views on testing female patients using CareStart™ G6PD RDT were also evaluated; pre-training, only 15.9% thought it was unreliable (combined score of 1–2) (Fig 5B). However, on Day 2 post-training, 78.5% correctly identified the kit as not suitable for female screening and the percentage increased to 93.4% by Week 8 (combined score of 1–2).

Pre-training, 44.6% trainees were undecided and with additional 2% of trainees ‘not comfortable’ (combined score of 1–2) with providing PQ based on RDT results alone. The percentage of trainees who were comfortable to guide their PQ treatment decision based on RDT results (combined score of 6–7) increased from 42.5% (baseline) to 88.7% post training on Day 2 (p < 0.001), with additional increase to 96% at week 8 (p < 0.001). There was no observed decrease in willingness to prescribe PQ with additional experience in the field (p = 0.102; Fig 5C). When given the option of G6PD screening with tests that are known to misclassify some patients, 33.7% of trainees reported it as non-acceptable (combined score of 1–2) but 47.9% of trainees were willing to use screening tests despite their known limitations (combined score of 6–7; Fig 5D). Post-training on Day 2, 53.0% of trainees reported that the likelihood of obtaining wrong result on RDT was low (combined score of 1–2). Post-training, the percentage of trainees reporting high likelihood (combined score of 6–7) of getting incorrect G6PD result with RDT was 26%, compared to 4% at baseline (p < 0.001; Fig 5E), but with no additional risk of misclassification with continued field experience (p = 0.197; Fig 5E). Despite trainees reporting that misclassifications of G6PD status can occur with RDTs, 95.9% were willing to prescribe PQ on Day 2 post training with 98.7% still willing to prescribe PQ after their continued experience in the field (Fig 5F).

## Discussion

No prior studies have evaluated healthcare community perceptions on PQ use, acceptability of G6PD screening with RDTs, and provider comfort level with prescribing PQ based on CareStart^TM^ G6PD test results, which is the point-of-care test recommended by the WHO in resource limited settings [19]. The study also assessed changes in perceptions about PQ risk following training and field experiences with the diagnostic test, and assessed trainee understanding of the limitations of G6PD screening. This study also established normal reference values of G6PD activities in Cambodian population based on the Point Scientific quantitative test.

Previous study from Cambodia reported a higher 100% G6PD activity value of 11.8 U/g Hb but results were based on a smaller cohort of non-randomly selected population [20]. Our study results from this larger cohort in Cambodia allow for a better estimate of the distribution of G6PD activities in male patients and helps establish thresholds of normal G6PD activity for quantitative tests in this region. The AMM for the study population (8.3 U/g Hb) is consistent with previous reports published for Myanmar where the adjusted median activity among the normal male population was 8.28 U/g Hb [21] and similar to the value of 8.7 U/g Hb among malaria infected patients in Cambodia [22]. Older studies from Cambodia reported much higher values for the AMM up to 12.6 U/g Hb [5, 14] which differed from other countries in Southeast Asia and it might have been due to differences in methodology or other unrecognized test limitations.

This is also the first study in Cambodia to evaluate the CareStart^TM^ G6PD RDT performance in the hands of the HCWs and VMWs in field settings, where it was demonstrated that the G6PD RDT performs well at the 30% cut-off activity, with test sensitivity and specificity in Cambodia higher than reported by others [20]. The healthcare staff who participated in this study had limited exposure to PQ and G6PD screening prior to the training workshop. Results indicate that trainees were more willing to screen patients for G6PD*d* and provide primaquine following 2-day workshop training. This was likely due to increased understanding of the diagnostic tests and the benefits and risks of PQ treatment. In addition, trainees had greater comfort level in RDTs with continued use in the field.

Despite demonstrated understanding of the test limitations, most trainees expressed willingness to incorporate testing into *Pv* case management, and were willing to accept the risks associated with treatment. Practice with known G6PD controls prior to evaluation in the field likely contributed to the high comfort level of using RDTs in the field in our study population. We cannot rule out the possibility that demonstrated positive results were influenced by the reluctance of an individual to give negative feedback; however, the volunteers willingly reported that RDTs have limitations, and the study conclusions are based not only on the individual perceptions about the test, but also based on improved knowledge scores and other measures of outcomes as presented. Access to radical cure treatment can be improved in Cambodia by making G6PD screening available at the point-of-care. However, it remains to be seen if VMWs or other healthcare workers will be given this new responsibility to screen for G6PD*d* and/or dispense PQ, which is not without risks. It is reassuring that with appropriate training and oversight, test performance in the field by novice users could match the performance from laboratory as previously reported (19). Yet, given the possible risks, the deployment of diagnostic tests may be more suitable at health centers, where oversight of G6PD screening can be more easily done. Deployment of RDTs will require significant resources to ensure quality control and oversight are in place.

There was greater tendency for trainees to over diagnose G6PD*d* compared to the expert technicians in the lab who had prior knowledge of RDTs. This is less of a concern when CareStart^TM^ RDT is introduced into clinical care when the emphasis is placed on minimizing the risks of hemolysis. However, this finding is of great importance to the operational deployment of RDTs in countries aiming to eliminate *P*.*vivax* malaria, since the diagnosis of G6PD deficiency will invariably result in patients being denied the radical cure. Patients who are classified as being deficient may benefit with repeat testing on malaria recurrence, or should be referred for confirmatory testing in certified laboratories. It should be highlighted that no group (trainee or expert study staff) was free from misclassifying G6PD*d*, highlighting the importance of the pharmacovigilance to monitor for adverse reactions from PQ. Access to blood products should be readily available at locations where PQ is introduced, in the event of misclassification and acute hemolysis requiring blood transfusion.

The presented findings highlight that G6PD screening can be effective post training even in the settings with limited resources. The majority of trainees expressed interest in implementation of RDTs despite perceived test limitations and their understanding of the risks and benefits from PQ treatment. It is possible that new point-of-care quantitative tests for G6PD*d* will have greater accuracy than the available RDTs, but this is yet to be demonstrated in the field studies. CareStart^TM^ G6PD RDT is currently the most promising point-of-care test, inexpensive and already available commercially. Future studies will need to evaluate its performance in the field against quantitative biosensors which require different skillset in test interpretation.

The demonstrated test sensitivity of >95% in detecting G6PD deficiency in male volunteers, and high negative predictive value exceeding 95%, meet the thresholds for a G6PD diagnostic test as recommended by the WHO [19]. The training was effective across all education levels and years of experience so could be widely adopted at different settings. The training on the test limitations did not affect negatively the willingness of the trainees to use the RDTs. The vast majority of trainees were willing to introduce PQ despite recognized risks and test limitations. This is likely due to recognized high burden of *Pv* in communities where training took place. The high acceptability of G6PD RDTs was maintained with continued experience in the field.

Our findings should be taken with caution prior to implementation of screening with available RDTs. CareStart™ RDTs can only be used in male patients and lack a control line. Special attention must be made to placement of the appropriate amount of blood using a pipette, and reading of the results must be at 10 minutes. Additional information is needed on how severe anemia might affect performance of G6PD RDTs since presence of reticulocytes might overestimate G6PD activity. The screening for anemia should be done with focused questions or measuring of baseline hemoglobin values as these patients have lower reserve for tolerating hemolysis. This will invariably result in higher cost of treatment.

Even though the questionnaires were translated into the local language (Khmer), some of the questions were viewed as technical and required clarification by the study staff. This might have introduced bias in the study results. The trainees also provided us with feedback that pictures included in the counseling script (S1 File) were adequate for communicating the key messages on PQ and G6PD screening, and were preferred to the text in the counseling script. The positive results might be different in settings where observers are not utilized. We tried to minimize the bias by blinding the trainees and experts to the results of quantitative tests. For a successful deployment of any G6PD screening test, ongoing supervision will be beneficial, to include periodic re-training and on-site spot checks. As an added measure of safety, formal certification in G6PD screening should be established. Healthcare providers who are given the responsibility to dispense or prescribe PQ should demonstrate adequate knowledge on risks and the test limitations to be able to effectively counsel patients.

## Conclusions: Implications for practice, policy and research

Increasing access to G6PD screening will be important to reach the goal for eliminating *Pv* malaria from Cambodia by 2025 [23]. CareStart^TM^ RDT can be offered at the point-of-care for male patients with *Pv* malaria to alleviate the lack of testing for G6PD*d* in rural settings. However, incorrect interpretation of G6PD results will occur in small percentage of patients. Therefore, safety monitoring and capacity building on how to diagnose and manage adverse events related to PQ must also be in place before wide deployment of PQ in Cambodia. Over diagnosis of G6PD deficiency will also occur when RDTs are deployed. Future tafenoquine (TQ) deployment will require a different set of tools to include testing with quantitative biosensors. These point-of-care quantitative tests will come with their own challenges in test interpretation and may not be suitable for all settings.

## Supporting information

S1 Table Checklist for performing G6PD screening test and recording of the results. (DOCX)

S2 Table Knowledge assessment pre- and post-training. (DOCX)

S1 File Patient counseling script. (DOCX)
