# Supplementary figures and images for: Evaluation of the CareStart™ glucose-6-phosphate dehydrogenase (G6PD) rapid diagnostic test in the field settings and assessment of perceived risk from primaquine at the community level in Cambodia
Source: PLoS One. 2020 Jan 31;15(1):e0228207. doi: 10.1371/journal.pone.0228207 (PMC6994100; doi:10.1371/journal.pone.0228207)

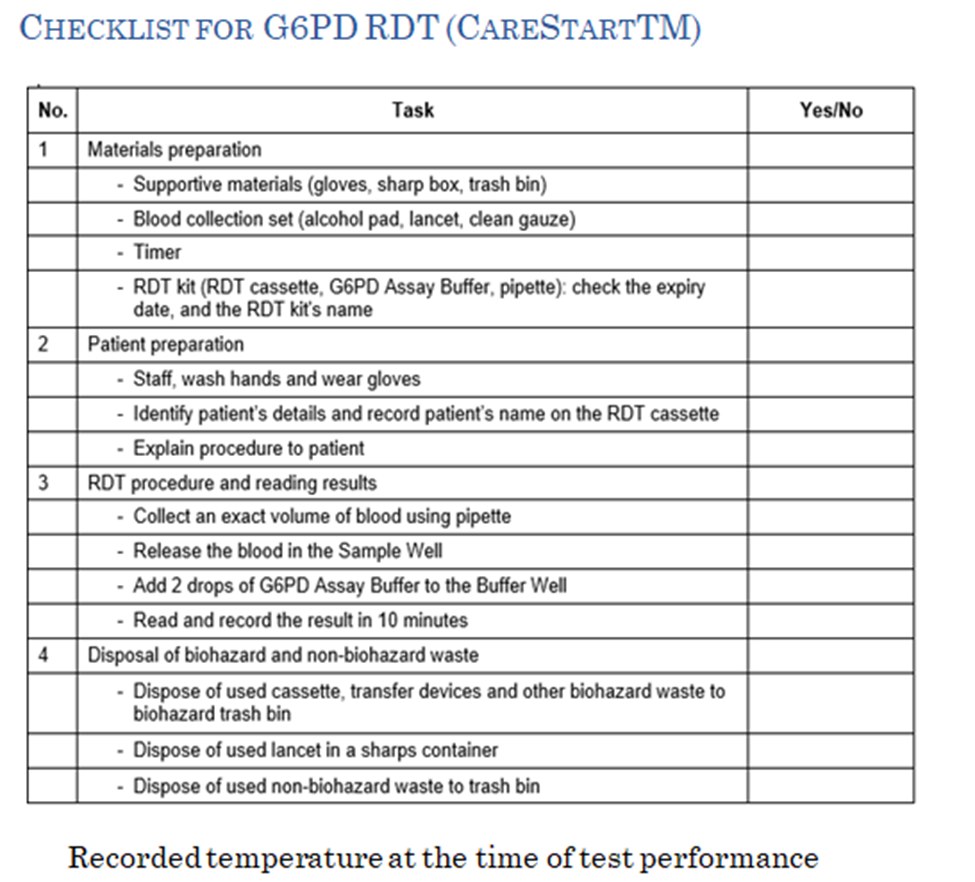
**S1 Table. Checklist for performing G6PD screening test and recording of the results**

Supplement: S1 Table — (DOCX) [file pone.0228207.s001.docx]
