# Supplementary material for: Evaluation of the CareStart™ glucose-6-phosphate dehydrogenase (G6PD) rapid diagnostic test in the field settings and assessment of perceived risk from primaquine at the community level in Cambodia
Source: PLoS One. 2020 Jan 31;15(1):e0228207. doi: 10.1371/journal.pone.0228207 (PMC6994100; doi:10.1371/journal.pone.0228207)
Supplement: S2 Table — (DOCX) [file pone.0228207.s002.docx]

| **S2 Table. Knowledge assessment pre- and post-training.** | **Concepts evaluated** | **Correct answer** | **Baseline** | **Day 2** | **p-value^a^** | **Week 2** | **p-value^a^** |
| --- | --- | --- | --- | --- | --- | --- | --- |
| **Do you know what primaquine is used for?**  (If yes, specify________________________) | Appropriate indications for use of primaquine | No  Yes  Missing data | 62 (61.39)  39 (38.61)  0 | 9 (8.91)  89 (88.12)  3 (2.97) | <0.001 | 7 (6.93)  90 (89.11)  4 (3.96) | 0.5930 |
|  |  |  |  |  |  |  |  |
|  |  |  |  |  |  |  |  |
| **Do you know of any benefits of primaquine for *P. vivax* malaria?** (If yes, specify__________________________) | Knowledge of the treatment benefit for *P.vivax* malaria | No  Yes  Missing data | 81 (80.20)  20 (19.80)  0 | 4 (3.96)  94 (93.07)  3 (2.97) | <0.001 | 4 (3.96)  93 (92.08)  4 (3.96) | 1.000 |
|  |  |  |  |  |  |  |  |
|  |  |  |  |  |  |  |  |
| **Do you know of any benefits of primaquine for *P. falciparum* malaria?** (If yes, specify_____________________________) | Knowledge of the treatment benefit for *P.falciparum* malaria | No | 81 (80.20) | 6 (5.94) | <0.001 | 5 (6.93) | 0.6547 |
|  |  | Yes | 20 (19.80) | 92 (91.09) |  | 92 (89.11) |  |
|  |  | Missing data | 0 | 3 (2.97) |  | 4 (3.96) |  |
| **Do you know of any risks or side effects associated with taking primaquine?** (If yes, specify___________________) | Knowledge of the side effects | No | 81 (80.20) | 6 (5.94) | <0.001 | 7 (6.93) | 0.7630 |
|  |  | Yes | 20 (19.80) | 92 (91.09) |  | 90 (89.11) |  |
|  |  | Missing data | 0 | 3 (2.97) |  | 4 (3.96) |  |
| **Primaquine should NOT be used when the patient has the following (select all that apply):**   Headache   Fever   Malaria   Mild abdominal pain   G6PD deficiency   I do not know | G6PD deficiency is a contraindication to primaquine use | No | 62 (61.39) | 16 (15.84) | <0.001 | 12 (11.88) | 0.3458 |
|  |  | Yes | 39 (38.61) | 82 (81.19) |  | 85 (84.16) |  |
|  |  | Missing data | 0 | 3 (2.97) |  | 4 (3.96) |  |
| **When taking primaquine, patient should be monitored for the following (select all that apply):**   Pallor  Dizziness Yellow skin or eyes   New onset back pain   New onset or worsening fatigue   Shortness of breath   Change in color of your urine to dark, cola color   Monitoring is NOT needed   I do not know | Patients require monitoring for signs and symptoms of hemolysis following the treatment with primaquine | No | 91 (90.10) | 17 (16.83) | <0.001 | 8 (7.92) | 0.0389 |
|  |  | Yes | 10 (9.90) | 81 (80.20) |  | 89 (88.12) |  |
|  |  | Missing data | 0 | 3 (2.97) |  | 4 (3.96) |  |
| **According to treatment guidelines, who should be treated with Primaquine** **(select all that apply):**   All patients with falciparum malaria (G6PD normal and G6PD deficient patients)   All patients with falciparum malaria and G6PD normal results   All patients with vivax malaria (G6PD normal and G6PD deficient patients)   All patients with vivax malaria and G6PD normal results   I do not know | Single dose of primaquibe for *P.falciparum* does not require screening for G6PD deficiency; whereas, patients with *P.vivax* must have normal G6PD status confirmed prior to receiving primaquine | No | 99 (98.02) | 44 (43.56) | <0.001 | 40 (39.60) | 0.5465 |
|  |  | Yes | 2 (1.98) | 54 (53.47) |  | 57 (56.44) |  |
|  |  | Missing data | 0 | 3 (2.97) |  | 4 (3.96) |  |
| **What is the benefit of treatment with primaquine (select all that apply):**   Primaquine kills the parasites that hide in the liver   Primaquine can lower the risk of having vivax malaria recurrence   Primaquine can be used for malaria treatment on its own without other drugs (as monotherapy)   I do not know | Benefits of treatment with primaquine include radical cure and lower risk of malaria recurrence from relapse. Primaquine cannot be used as monotherapy. | No | 88 (87.13) | 14 (13.86) | <0.001 | 17 (16.83) | 0.5775 |
|  |  | Yes | 13 (12.87) | 84 (83.17) |  | 80 (79.21) |  |
|  |  | Missing data | 0 | 3 (2.97) |  | 4 (3.96) |  |
| **What may happen if patient accidentally takes Primaquine when he/she is G6PD deficient?**   Patient may become sick from the side effects of primaquine   Patient may develop weakness or sudden change in the color of the urine   Red blood cells that carry oxygen can get damaged   Nothing will happen because primaquine is very safe   I do not know | Primaquine can cause signs and symptoms of hemolysis | No | 88 (87.13) | 34 (33.66) | <0.001 | 19 (18.81) | 0.0112 |
|  |  | Yes | 13 (12.87) | 64 (63.37) |  | 78 (77.23) |  |
|  |  | Missing data | 0 | 3 (2.97) |  | 4 (5.26) |  |
| **The risk of side effects with primaquine can be reduced with G6PD testing:**   True  False  I do NOT know | G6PD screening prior to treatment reduces the risk of side effects (acute hemolysis) | No | 54 (53.47) | 2 (1.98) | <0.001 | 3 (2.97) | 0.6547 |
|  |  | Yes | 47 (46.53) | 96 (95.05) |  | 94 (93.07) |  |
|  |  | Missing data | 0 | 3 (2.97) |  | 4 (3.96) |  |
| **Primaquine is recommended for cure in patients with vivax malaria if they have normal G6PD test results:**   True  False  I do NOT know | Primaquine treatment is recommended for *P.vivax* malaria in patients with normal G6PD status | No | 51 (50.50) | 0 | <0.001 | 1 (0.99) | 0.3173 |
|  |  | Yes | 50 (49.50) | 97 (96.04) |  | 96 (95.05) |  |
|  |  | Missing data | 0 | 4 (3.96) |  | 4 (3.96) |  |
| **Primaquine is required to be cured from falciparum malaria:**   True  False  I do not know | Primaquine use in patients with *P.falciaprum* is for transmission blocking  and is not required for cure | No | 86 (85.15) | 42 (41.58) | <0.001 | 42 (41.58) | 1.0000 |
|  |  | Yes | 14 (13.86) | 56 (55.45) |  | 55 (54.46) |  |
|  |  | Missing data | 1 (0.99) | 3 (2.97) |  | 4 (3.96) |  |
| **If a patient has falciparum malaria, they can take 1 dose of primaquine without checking their G6PD status.**   True  False  I do NOT know | No G6PD screening is required for taking single dose primaquine | No | 66 (65.35) | 1 (0.99) | <0.001 | 1 (0.99) | 1.0000 |
|  |  | Yes | 35 (34.65) | 97 (96.04) |  | 96 (95.05) |  |
|  |  | Missing data | 0 | 3 (2.97) |  | 4 (3.96) |  |
| **Who should be tested for G6PD deficiency using the Rapid Diagnostic Test?**   Males  Females   Both males and females   I don’t know | G6PD RDTs can only reliably screen male patients | No | 32 (31.68) | 1 (0.99) | <0.001 | 0 | 0.3173 |
|  |  | Yes | 69 (68.32) | 97 (96.04) |  | 97 (96.04) |  |
|  |  | Missing | 0 | 3 (2.97) |  | 4 (3.96) |  |
| **How will you prescribe primaquine for adult patient with vivax malaria (G6PD normal):**   14 tablets to take in one day   One tablet taken every day, until patient feels better   1 tablet a day for 14 days to complete full 2 week course, even if all malaria symptoms disappear at the end of week one   1 tablet on the first day of treatment only   I do not know | National treatment guidelines recommend 2 weeks treatment for *P.vivax* in G6PD normal individuals (15 mg tablet once a day for 2 weeks) | No | 65 (64.36) | 11 (10.89) | <0.001 | 14 (13.86) | 0.4669 |
|  |  | Yes | 36 (35.64) | 87 (86.14) |  | 83 (82.18) |  |
|  |  | Missing data | 0 | 3 (2.97) |  | 4 (3.96) |  |
| **After I take primaquine for vivax malaria, which of the following is true:**   I can still get vivax malaria again if I get bitten by an infected mosquito in the future   I am permanently protected from vivax malaria after I take Primaquine   I do not know | Despite radical cure, primaquine does not prevent re-infections | No | 78 (77.23) | 10 (9.90) | <0.001 | 11 (10.89) | 0.7630 |
|  |  | Yes | 23 (22.77) | 88 (87.13) |  | 86 (85.15) |  |
|  |  | Missing data | 0 | 3 (2.97) |  | 4 (3.96) |  |
| **Taking 1 dose of primaquine when patient has falciparum malaria helps the community by lowering the risk of malaria spread.**   True  False  I do NOT know | Single dose of primaquine is effective and beneficial for blocking *P.falciparum* malaria transmission in the community | No | 54 (53.47) | 2 (1.98) | <0.001 | 2 (1.98) | 1.0000 |
|  |  | Yes | 47 (46.53) | 96 (95.05) |  | 95 (94.06) |  |
|  |  | Missing data | 0 | 3 (2.97) |  | 4 (3.96) |  |
| **Taking 1 dose of primaquine by a patient with falciparum malaria is generally safe for patients irrespective of their G6PD status (normal and G6PD deficient).**   True  False  I do NOT know | No screening for G6PD deficiency is required for single dose of primaquine to block  transmission of *P.falciparum* malaria | No | 75 (74.26) | 2 (1.98) | <0.001 | 2 (1.98) | 1.0000 |
|  |  | Yes | 26 (25.74) | 96 (95.05) |  | 95 (94.06) |  |
|  |  | Missing data | 0 | 3 (2.97) |  | 4 (3.96) |  |
| **Taking 2 week course of primaquine by a patient with vivax malaria is safe, without G6PD testing.**   True  False  I do NOT know | G6PD testing is recommended prior to taking 2-week course of  primaquine for *P.vivax* malaria | No | 57 (56.44) | 6 (5.94) | <0.001 | 9 (8.91) | 0.3657 |
|  |  | Yes | 44 (43.56) | 92 (91.09) |  | 87 (86.14) |  |
|  |  | Missing data | 0 | 3 (2.97) |  | 5 (4.95) |  |
| **If urine suddenly becomes dark, cola-colored (shown below), the patient should:**  Seek medical care immediately   Monitor this situation over the next 3-5 days   Continue taking primaquine to make sure can be cured from malaria   I do NOT know | Change in the color of urine can be a sign of hemolysis that requires immediate medical care | No | 65 (64.36) | 4 (3.96) | <0.001 | 5 (4.95) | 0.5637 |
|  |  | Yes | 36 (35.64) | 94 (93.07) |  | 92 (91.09) |  |
|  |  | Missing data | 0 | 3 (2.97) |  | 4 (3.96) |  |
| **Pregnant women and women who are breastfeeding can take primaquine safely.**   True  False  I do NOT know | Knowledge of contraindications to treatment with primaquine (i.e pregnancy) | No | 45 (44.55) | 3 (2.97) | <0.001 | 0 | 0.0833 |
|  |  | Yes | 56 (55.45) | 95 (94.06) |  | 97 (96.04) |  |
|  |  | Missing data | 0 | 3 (2.97) |  | 4 (3.96) |  |
| **What is the benefit of primaquine treatment for a patient with *P.falciparum*?**   No benefit, treatment is NOT recommended   Benefit is small   The community can benefit by lowering the risk of malaria transmission to others   I do NOT know | The community can benefit by lowering the risk of malaria transmission when primaquine is used for treatment of *P. falciparum* malaria | No | 51 (50.50) | 1 (0.99) | <0.001 | 3 (2.97) | 0.1573 |
|  |  | Yes | 50 (49.50) | 97 (96.04) |  | 94 (93.07 |  |
|  |  | Missing data | 0 | 97 (96.04) |  | 4 (3.96 |  |
| **How beneficial is primaquine treatment for patients with *P.vivax*?**   No benefit, treatment is NOT recommended   Benefit is small   Majority of patients can benefit from treatment (it is required for cure)   I do NOT know | Majority of patients who have *P. vivax* can benefit from treatment with primaquine (it is required for radical cure). | 0. No | 57 (56.44) | 2 (1.98) | <0.001 | 3 (2.97) | 0.6547 |
|  |  | 1. Yes | 44 (43.56) | 96 (95.05) |  | 94 (93.07) |  |
|  |  | Missing data | 0 | 3 (2.97) |  | 4 (3.96) |  |
| **How long do you have to wait before reading G6PD RDT results (CareStart^TM^)?**   2 min  5 min  10 min   15 min  I don’t know | Reading of the RDT must be done at 10 mins | No | 89 (88.12) | 0 | <0.001 | 0 | 1.0000 |
|  |  | Yes | 12 (11.88) | 98 (97.03) |  | 97 (96.04) |  |
|  |  | Missing data | 0 | 3 (2.97) |  | 4 (3.96) |  |
| **What can affect the test performance of G6PD screening test such as CareStart^TM^?**  (select all that apply)   Temperature >32 degrees   Novice user not familiar with the color interpretation   Reading of the test at 15 mins rather than 10 mins   I do NOT know | Knowledge of test limitations | No | 98 (97.03) | 44 (43.56) | <0.001 | 36 (35.64) | 0.2579 |
|  |  | Yes | 3 (2.97) | 54 (53.47) |  | 61 (60.40) |  |
|  |  | Missing data | 0 | 3 (2.97) |  | 4 (3.96) |  |
| **When interpreting the G6PD RDTs, the patient is G6PD deficient based on following:** (select all that apply)   The color in the reading window is WHITE   The color in the reading window is PURPLE   The color in the reading window is VERY FAINT purple   I do NOT know | Able to diagnose G6PD deficiency based on color changes in the reading window of an RDT | No | 100 (99.01) | 69 (68.32) | <0.001 | 54 (53.47) | 0.0112 |
|  |  | Yes | 1 (0.99) | 29 (28.71) |  | 43 (42.57) |  |
|  |  | Missing data | 0 | 3 (2.97) |  | 4 (3.96) |  |
| **A male patient has G6PD result shown below, what do you recommend next?** (Picture of invalid RDT result shown)   Treatment with primaquine   Counsel the patient that he/she is G6PD deficient   Repeat the test   I don’t know | Correct recommendation on primaquine use according to the RDT results. Able to recognize when the RDT needs to be repeated due to invalid result. | No | 8 (7.92) | 16 (15.84) | <0.001 | 18 (17.82) | 0.6698 |
|  |  | Yes | 93 (92.08) | 82 (81.19) |  | 79 (78.22) |  |
|  |  | Missing data | 0 | 3 (2.97) |  | 4 (3.96) |  |
| **A male patient has G6PD result shown below, would you recommend primaquine?** (Picture of invalid RDT result shown) | Correct recommendation on primaquine use according to the RDT results. Knows when the result is invalid. | No | 92 (91.09) | 15 (14.85) | <0.001 | 22 (21.78) | 0.1615 |
|  |  | Yes | 9 (8.91) | 83 (82.18) |  | 74 (73.27) |  |
|  |  | Missing data | 0 | 3 (2.97) |  | 5 (4.95) |  |
| **A patient comes to the clinic and informs you that she is feeling tired and her lips look pale after taking her husband’s anti-malaria treatment. On inquiry, you discover her husband was prescribed primaquine and felt better quickly, so he shared his remaining medications with his wife. What is the most important step you should take at this time?**   Prescribe additional tablets of primaquine so the patient has enough to finish 2 weeks course   Discharge home with instruction to return if she develops a fever   Immediately refer to the nearest hospital for urgent evaluation   I do NOT know | Knows appropriate management of suspected acute  hemolysis/anemia (referral to higher level of care for evaluation is warranted) | No  Yes  Missing data | 54 (53.47)  47 (46.53)  0 | 4 (3.96)  94 (93.07)  3 (2.97) | <0.001 | 4 (3.96)  93 (92.08)  4 (3.96) | 1.0000 |
|  |  |  |  |  |  |  |  |
|  |  |  |  |  |  |  |  |

^a^Stuart-Maxwell test was used to assess for the statistical differences in the knowledge at baseline vs. Day 2 post-training and Week 2 vs. Day 2 follow up.

There were a total of 29 knowledge questions, each correctly answered question was assigned a score of 1 and incorrectly answered questions received a score of 0. The table shows the number of volunteers (and percentage) who correctly answered each knowledge question. For multiple choice questions that had more than one correct answer, the volunteers had to select all correct statements to receive the credit and no partial credit was awarded. Significant improvements in knowledge of G6PD deficiency, test interpretation, and appropriate primaquine use was observed following training and volunteers retained the information on week 2 follow up. The identified gaps in training were as follows: primaquine is not required for cure of *P.falciparum*; environmental factors might have effect on test performance; and the interpretation of the color change for G6PD status determination. The low score for result interpretation based on the color change was largely due to misclassifications of the “very faint” color. With additional experience during the workshop, the knowledge scores for interpreting the color change improved.
