# Supplementary material for: Evaluation of the CareStart™ glucose-6-phosphate dehydrogenase (G6PD) rapid diagnostic test in the field settings and assessment of perceived risk from primaquine at the community level in Cambodia
Source: PLoS One. 2020 Jan 31;15(1):e0228207. doi: 10.1371/journal.pone.0228207 (PMC6994100; doi:10.1371/journal.pone.0228207)
Supplement: S1 File — (DOCX) [file pone.0228207.s003.docx]

**S1 File. Patient Counseling Script**

**Patient Counseling Script on G6PD deficiency and primaquine use**

**Prepared by:**

Bertha Wojnarski

Mariusz Wojnarski

Sabaithip Sriwichai

Chanthap Lon

Threechada Boonchan (graphics)

**INFORMATION ABOUT PRIMAQUINE & G6PD SCREENING**

**G6PD deficiency**

Some people have a low amount of a substance called glucose-6-phosphate dehydrogenase (G6PD) in the cells that carry oxygen in the blood around the body. These people have what is called G6PD deficiency. Most people with low G6PD levels never know they are different, and they do not show symptoms unless they take certain medications, such as Primaquine.


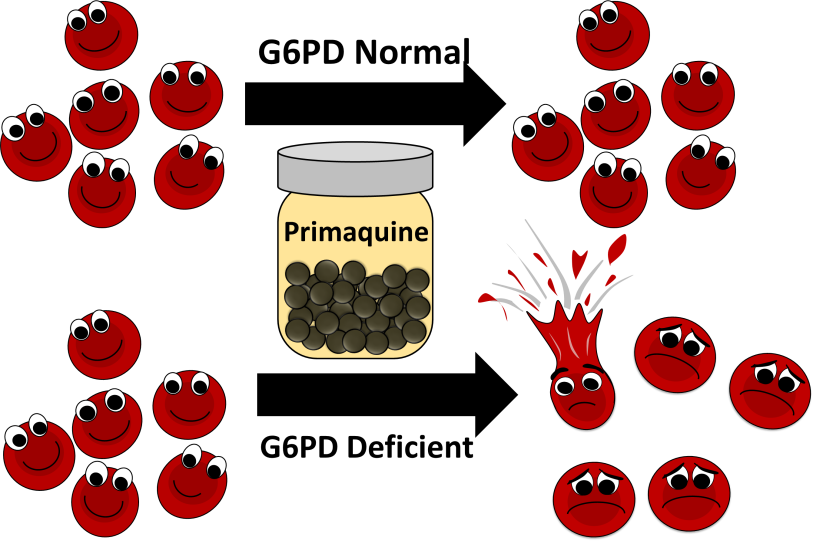


If the patient has a low amount of G6PD, their red blood cells can get broken or get damaged more easily.


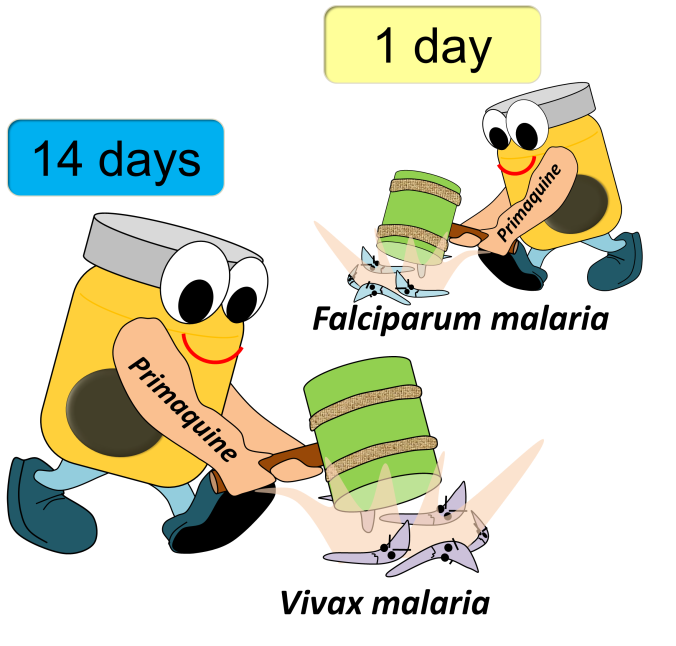


**Current malaria treatment recommendations in Cambodia**

Current treatment guidelines in Cambodia recommend that all malaria patients receive antimalarial drugs called artemisinin-based combination therapy (ACT) for 3 days. Patients with malaria should also receive treatment with Primaquine, as long as they have normal levels of G6PD in their red blood cells.

It is recommended that G6PD normal patients who have vivax malaria receive treatment with Primaquine every day for 14 days. Patients who have falciparum malaria should receive a single dose of Primaquine.

**
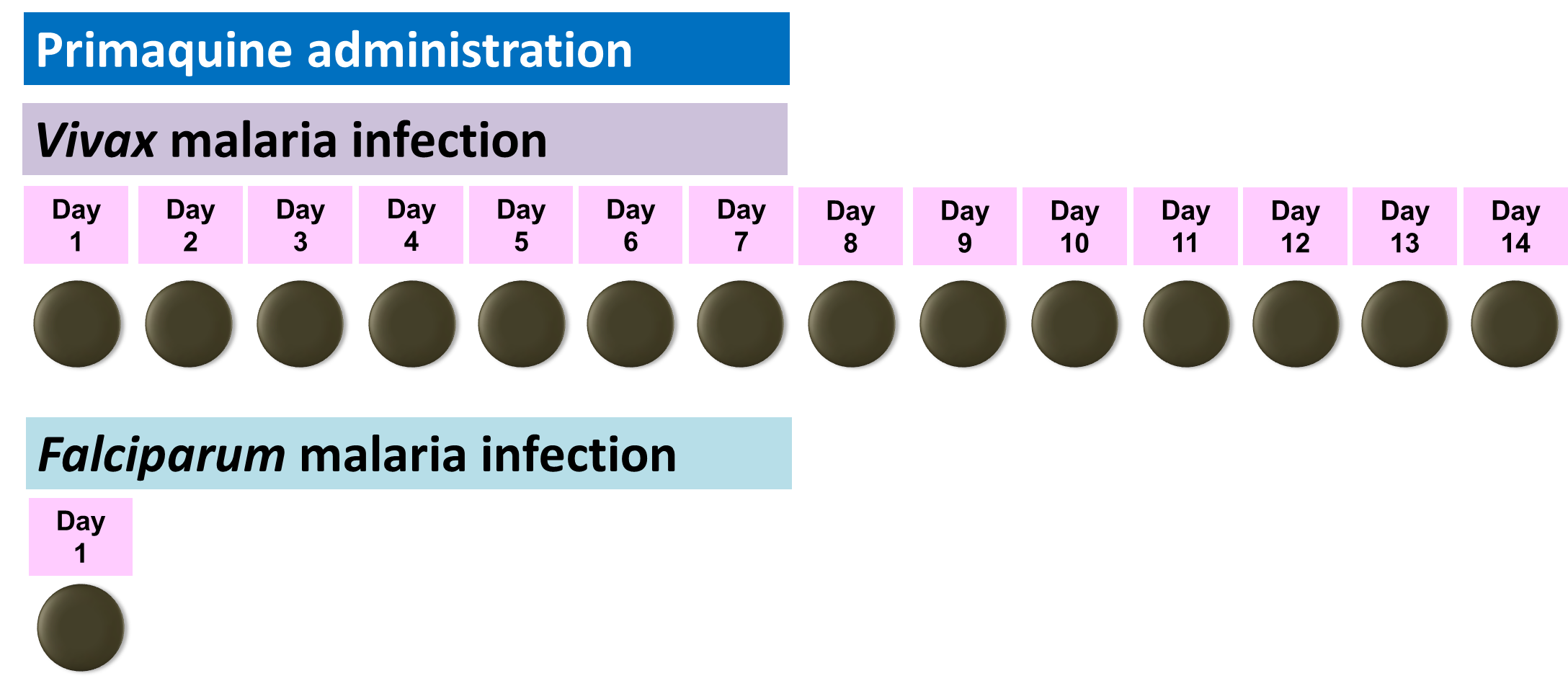
**

**Why is Primaquine recommended**

**for malaria treatment?**

If a patient has vivax malaria and does NOT get treatment with Primaquine, they can have parasites that stay hidden in the liver and enter blood several months later, which will make the patient sick again with malaria symptoms.

To get rid of these parasites from the liver, the patient needs to take Primaquine.


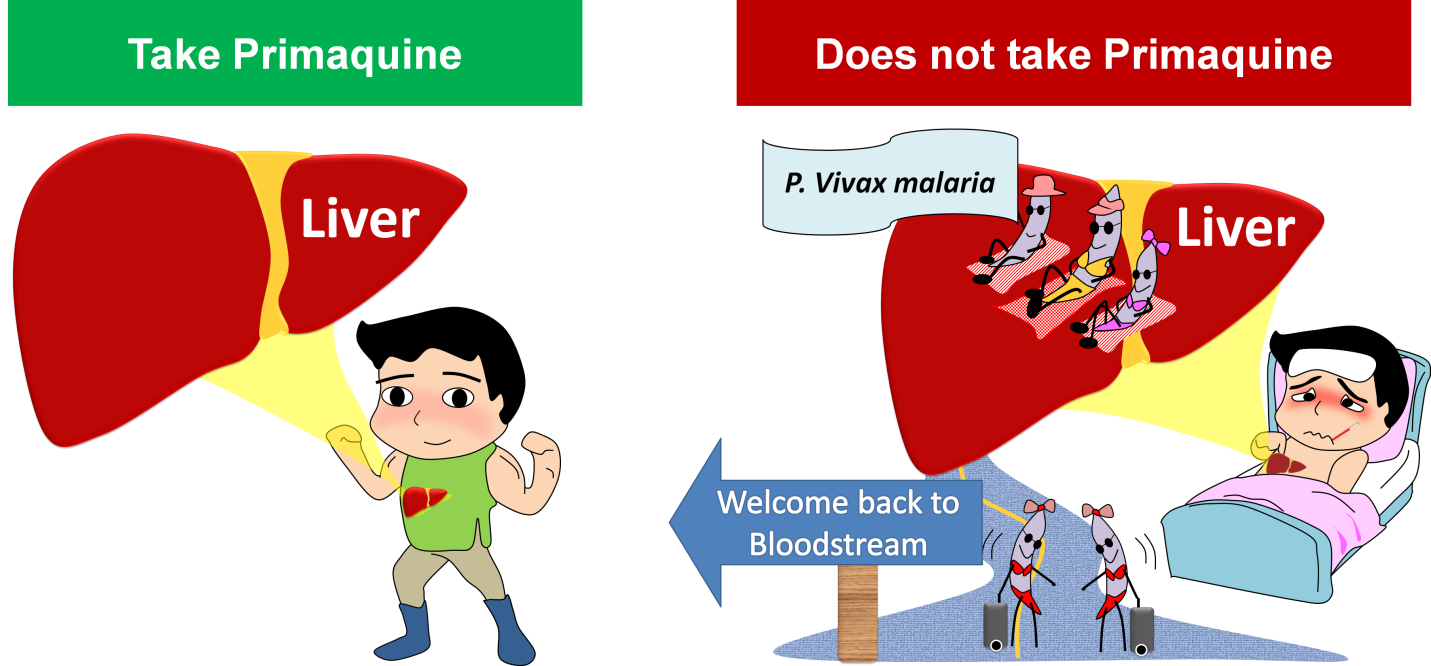


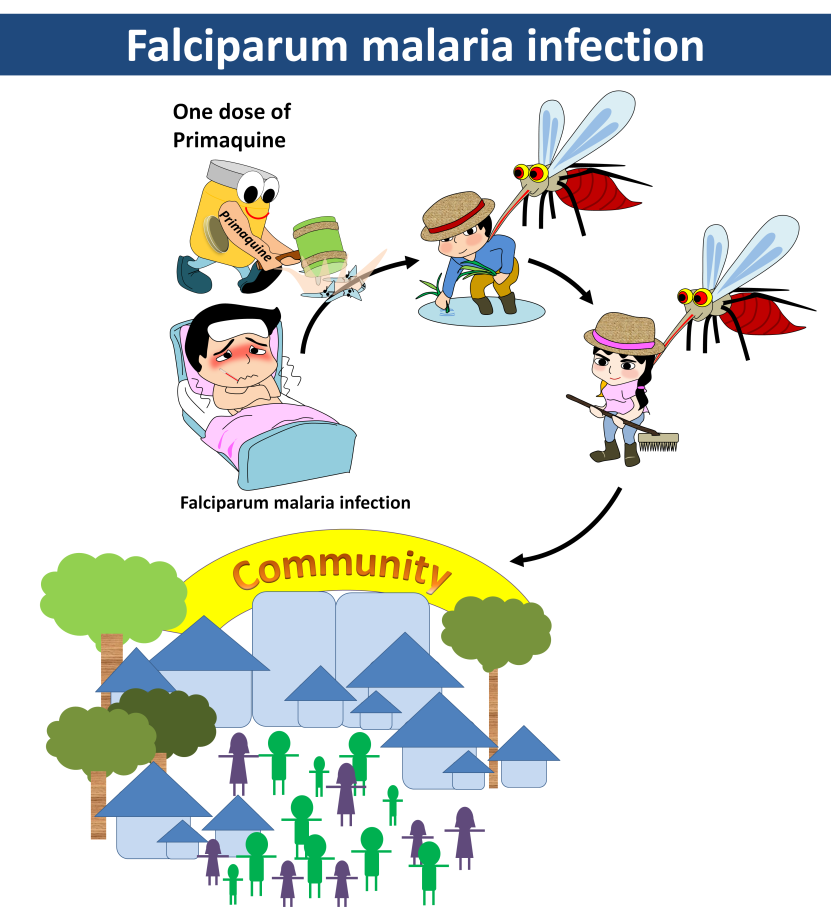


For patients who have **falciparum** malaria, it is recommended that they take **one dose** of Primaquine in order to reduce the risk of transmission of malaria to other people. This means fewer people will be able to transmit malaria in the community, so the risk of getting malaria when they get bitten by a mosquito may be **lower**.


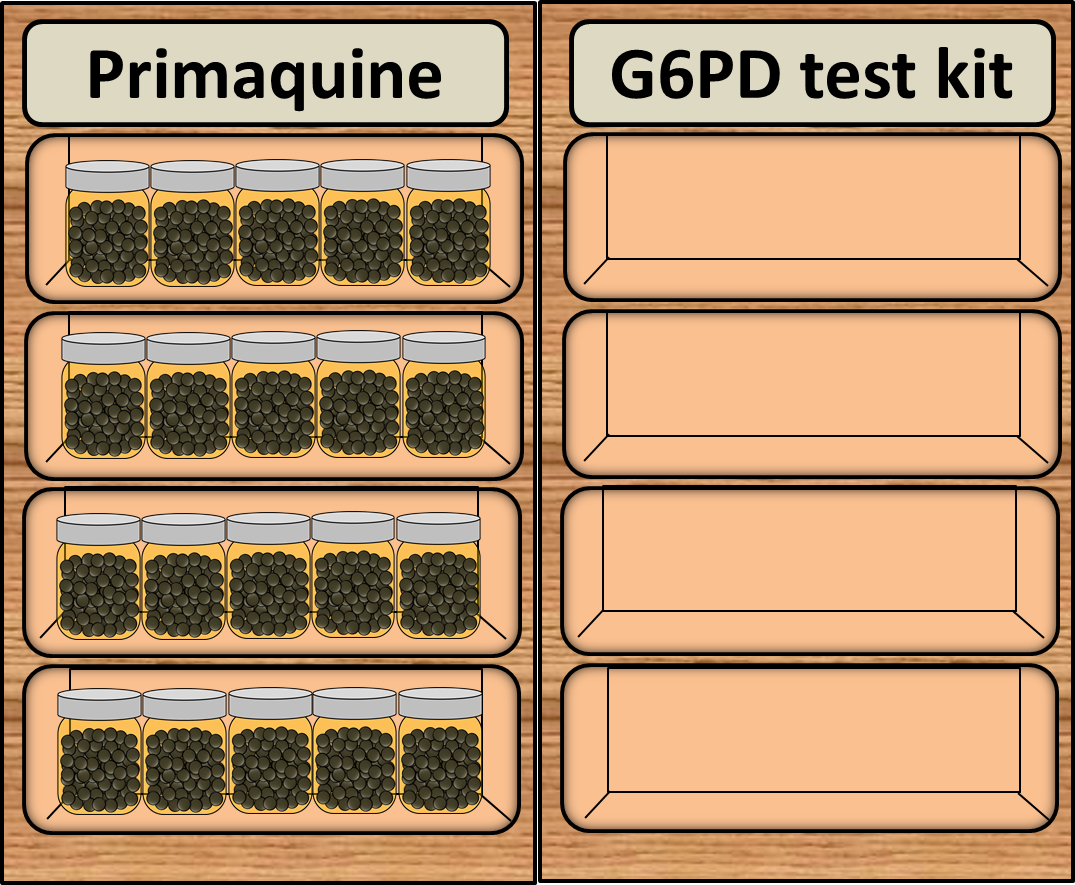

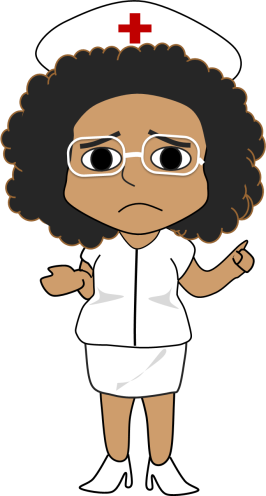


G6PD testing was not available in Cambodia, so Primaquine was not used*,* but the National Malaria Program (CNM) would like to make testing and Primaquine more widely available for malaria patients, in support of Cambodia malaria elimination strategy.

**What are the risks of treatment with Primaquine?**

For patients with NORMAL G6PD levels, taking Primaquine is generally very well tolerated and most people are able to complete the full course. You can take it with food to reduce stomach irritation.

But taking Primaquine is NOT recommended for people with G6PD deficiency, as the breakdown of the red blood cells can make them ill. The most common symptoms are fatigue, shortness of breath after activity, rapid breathing, pallor, and increased heart rate, yellowing of skin and eyes, or dark, tea colored urine or back pain. In most cases, this can be corrected by stopping Primaquine treatment when these symptoms develop. **Patients with NORMAL G6PD levels are very unlikely to develop these symptoms with Primaquine.**

If someone’s **G6PD levels are very low**, Primaquine treatment could result in a large number of their red blood cells being damaged. Therefore, in rare cases, some patients treated with Primaquine may need a blood transfusion to replace the red blood cells that were damaged after Primaquine treatment. This is very unlikely for patients with normal G6PD levels


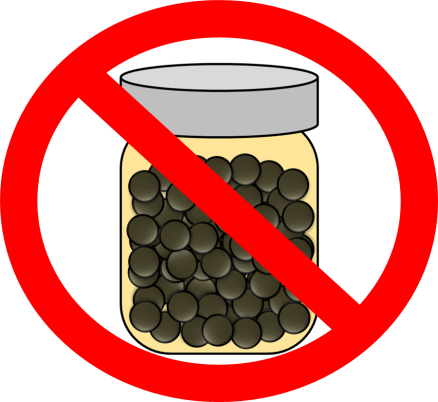


**Give ATC**

**Dose not give PQ**

**STOP Primaquine**


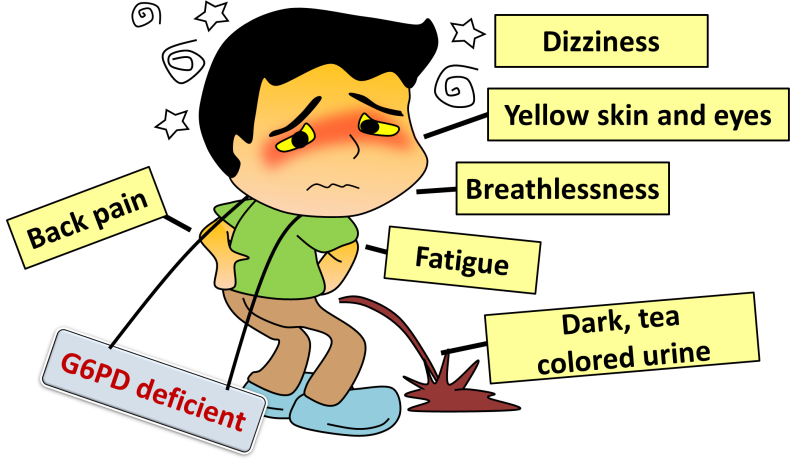


The risks of Primaquine treatment are based on patient’s G6PD level, how many doses of Primaquine they take and other factors. **Risk can be minimized if Primaquine is given only to patients with normal or near normal levels of G6PD. Therefore, testing for G6PD is recommended prior to treatment with Primaquine**

The risk from Primaquine treatment is also considered very low for falciparum patients since they only have to take a single dose. Single dose treatment is unlikely to result in significant destruction of red blood cells, even for patients with low levels of G6PD. **It is therefore acceptable to take SINGLE dose of Primaquine for falciparum malaria without G6PD testing.**


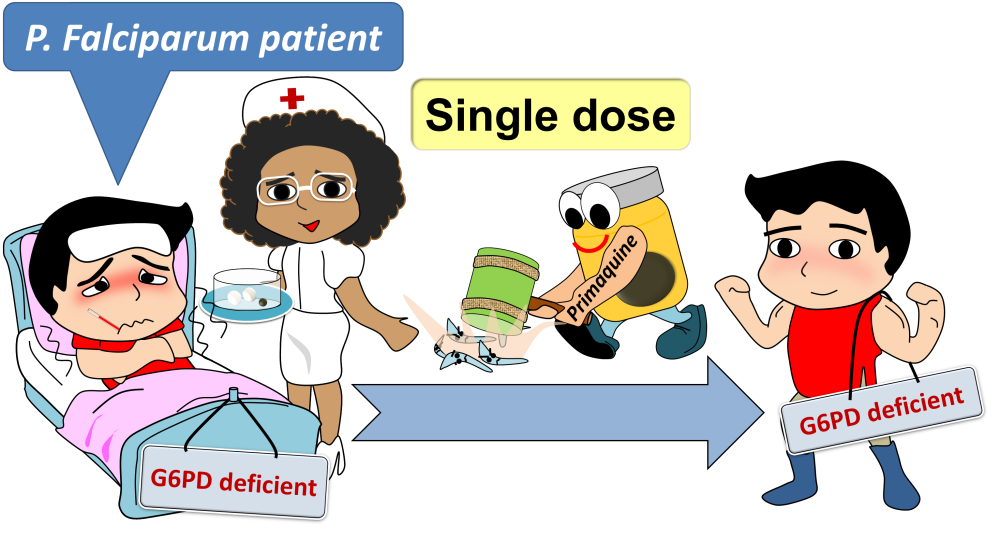


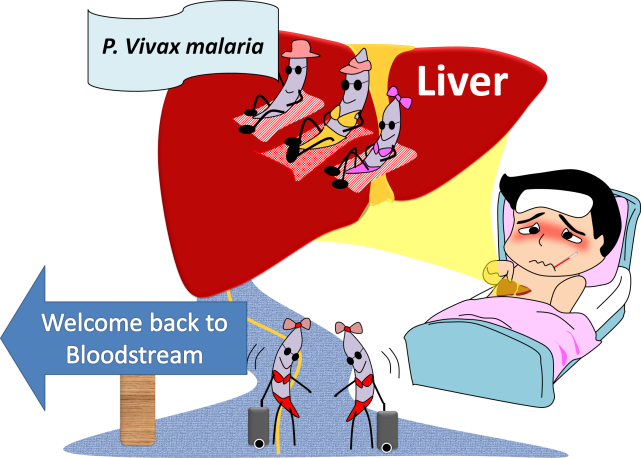


Lack of treatment with Primaquine in patients with vivax malaria also poses risks to patients as this can result in frequent recurrences of vivax malaria. Having malaria recurrence frequently can make your body weaker due to lowering of the red blood cells in your body, and it can affect your ability to work or take care of your family when you are sick.

**What are the benefits of PQ treatment?**

It is suspected that many patients have *vivax* recurrence due to lack of treatment with Primaquine, which allows the hidden parasites in the liver to enter the patient’s blood many times. Taking Primaquine when a patient is diagnosed with *vivax* will kill and eliminate hidden parasites in their liver and make them less likely to have recurrence of the infection. However, it is still possible for a patient to get vivax in the future if they get bitten again by an infected mosquito.

Taking Primaquine when a patient has *falciparum* malaria will make them less likely to transmit the infection to other people, which may reduce their own risk of getting infected again.

**Limitations of the current tests for G6PD screening:**

There are different tests for G6PD deficiency but most of them are not suitable for use outside of a highly specialized laboratory setting. The test for G6PD deficiency which is being used today comes with limitations as outlined below:

1. **
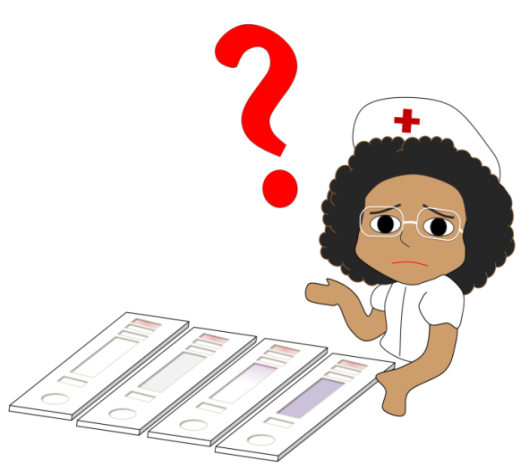
**The rapid diagnostic test for G6PD deficiency requires interpretation of a color change, which can sometimes be difficult, or color change can be misinterpreted even by highly trained medical staff.
2. The reliability of the test may be compromised if the testing is done in the room with more than 32 degrees Celsius.
3. The rapid diagnostic test can only tell if a patient has NORMAL or LOW levels of G6PD. These tests cannot tell how much of the G6PD enzyme the patient has.

**Therefore, it is recommended that even with NORMAL G6PD test result, all patients are monitored for possible side effects from taking Primaquine.**


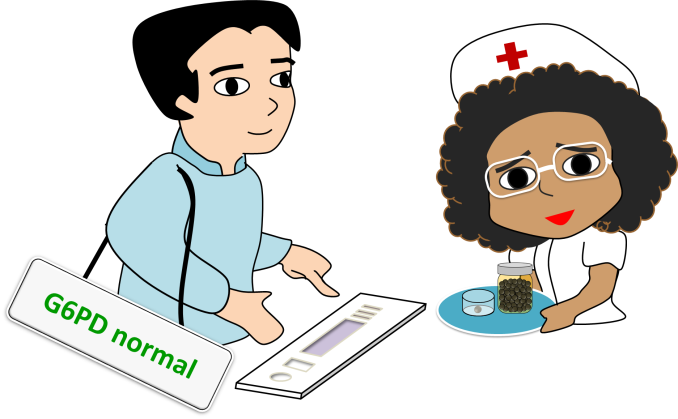
**Understanding your test results:**

If your test result is **normal** it means you should have sufficient levels of the G6PD enzyme to receive Primaquine safely

If the test shows you are **G6PD deficient**, this means that taking Primaquine for 14 days could damage some of your red blood cells and might make you feel sick. This can manifest as you being pale, having shortness of breath with activity, having a yellow pigmentation of the skin or eyes, or dark, tea-colored urine. So treatment with Primaquine is not recommended for vivax malaria when you are G6PD deficient. However, single dose of Primaquine for falciparum malaria is normally tolerated even in patients with G6PD deficiency; therefore, Primaquine is recommended to reduce risk of transmission to others when you have P. falciparum malaria.


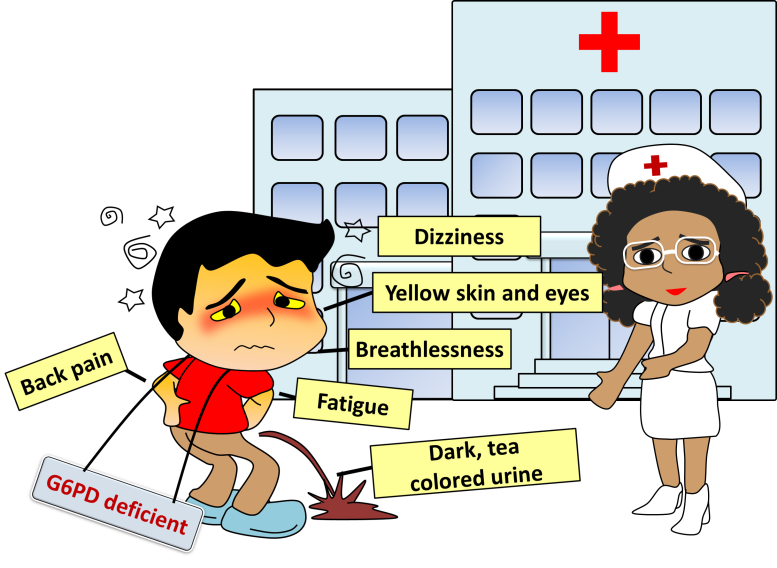


Given the limitations of the tests, if you are given Primaquine and develop the above mentioned symptoms or any new symptoms within 2 weeks of taking Primaquine, you should visit your doctor immediately. If your local health center is closed, then you should go to the nearest hospital.

**NOTE: If you were tested for G6PD deficiency under this pilot study, we will confirm the results of your rapid diagnostic test, with another G6PD test, which measures the actual amount of G6PD activity. This test needs to be performed in a highly specialized laboratory so the results will not be available immediately.**

**Rapid diagnostic testing for G6PD is appropriate only in male patients. Female patients require use of a test which provides actual value of G6PD activity but this specialized test may not be readily available at this time.**
